# Potential Role of lncRNA H19 as a Cancer Biomarker in Human Cancers Detection and Diagnosis: A Pooled Analysis Based on 1585 Subjects

**DOI:** 10.1155/2019/9056458

**Published:** 2019-03-19

**Authors:** Yuhan Liu, Anbang He, Baoer Liu, Zhengxian Huang, Hongbing Mei

**Affiliations:** ^1^Department of Urology, Shenzhen Second People's Hospital, the First Affiliated Hospital of Shenzhen University, Shenzhen 518000, China; ^2^Department of Urology, Peking University First Hospital, The Institute of Urology, Peking University, National Urological Cancer Centre, Beijing 100034, China; ^3^Department of Breast Surgery, Shenzhen Second People's Hospital, the First Affiliated Hospital of Shenzhen University, Shenzhen 518000, China

## Abstract

Long noncoding RNAs (lncRNAs) have been reported to serve as diagnostic and prognostic biomarkers of cancers, which play vital roles in tumorigenesis and tumor progression. Several studies have been performed to explore diagnostic value of lncRNA H19 in cancer detection and diagnosis. However, there are still inconsistent results in diagnostic accuracy and reliability in individual studies. Therefore, the present study was performed to summarize the overall diagnostic performance of lncRNA H19 in cancer detection and diagnosis. A total of eight studies with 770 cases and 815 controls were included in this pooled analysis. The pooled diagnostic results were as follows: sensitivity, 0.69 (95%CI=0.62-0.76), specificity, 0.79 (95% CI=0.70-0.86), positive likelihood ratio (PLR), 3.31 (95%CI=2.29-4.78), negative likelihood (NLR), 0.39 (95%CI=0.31-0.49), diagnostic odds ratio (DOR), 8.53 (95%CI=4.99-14.60), and area under the curve (AUC), 0.79 (95%CI=0.76-0.83). Deeks' funnel plot asymmetry test (P=0.13) suggested no potential publication bias. Our results indicated that lncRNA H19 had a relatively moderate accuracy in cancer detection and diagnosis. Further comprehensive prospective studies with large sample sizes are urgently required to validate our findings.

## 1. Introduction

With incremental incidence and mortality in recent years, cancer has been a major public health problem all over the world [1, 2]. Although tremendous improvements have been made in therapeutic method including surgery, radiotherapy, chemotherapy, and precision therapy over past decades, the prognosis and quality of life of cancer patients remain poor, particularly in patients with advanced staged or metastatic cancers [3–5]. The lack of early diagnostic techniques contributes to the current situation [2]. Therefore, finding a potential diagnostic biomarker with good specificity and sensitivity for early cancer detection and diagnosis seems urgently needed.

Long noncoding RNAs (LncRNAs) are a subclass of regulatory ncRNAs longer than 200 nucleotides, lacking functional open reading frames (ORFs) and protein-coding capability [6, 7]. LncRNAs are widely reported to regulate gene expression at epigenetic, transcriptional, and posttranscriptional levels [8–10] and aberrant expression of lncRNAs can be involved in cancer initiation, progression, and metastasis [11–13]. Furthermore, increasing evidences suggested that lncRNAs could serve as potential biomarkers with high sensitivity and specificity in cancer detection and diagnosis [14–17].

H19, a subclass of long noncoding RNA, is a paternally imprinted gene which locates in chromosome 11p15.5 [18]. In recent years, lncRNA H19 was identified to be significantly associated with various human cancers including breast cancer, gastric cancer, thyroid cancer, and hepatic carcinoma [19–26]. Several studies indicated that lncRNA H19 could function as an oncogene in tumorigenesis and tumor progression [27–29]. Quite a few studies had explored the clinical use of lncRNA H19 in cancer detection and diagnosis. However, the diagnostic accuracy of lncRNA H19 in the individual studies is still inconsistent and controversial. For example, Zhou et al. [23] showed that lncRNA H19 can be used for diagnosis of gastric cancer with a moderate-high sensitivity and specificity of 82.9% and 72.9%, respectively, but Hashad et al. [20] revealed a low sensitivity and specificity of 68.75% and 56.67%, respectively, in gastric cancer detection. These results failed to reach the agreement due to the difference of ethnicity, study design, types of tumors, stage of cancer, and the small sample size, which made it difficult to interpret. Thus, this pooled analysis was conducted to summarize the overall diagnostic performance of lncRNA H19 in cancer detection and diagnosis and further explored its clinical value.

## 2. Methods

### 2.1. Search Strategy and Study Selection Criteria

Literature research was performed in database including PubMed, Web of Science, Wanfang library, and CNKI up to May 18, 2018, by the following searching strategy: “cancer” or “tumor” or “carcinoma” or “neoplasm” or “malignancy” or “neoplasm” and “H19” and “sensitivity” or “specificity” or “ROC curve” or “accuracy”. Three investigators (HAB, LBE, and LYH) checked the titles and abstracts of the studies and scanned the full texts to eliminate irrelevant studies with the following included criteria: (1) the diagnostic value of lncRNA H19 for detecting cancer evaluated in articles, (2) explicitly defined article population and control sources; (3) completed data for calculating sensitivity and specificity; and (4) being published in English or Chinese.

### 2.2. Data Extraction and Quality Assessment

For each study, the following information was extracted: first author, year of publication, country, ethnicity, sample size, specimen and cancer type, detection method, cutoff value, true positive (TP), false positive (FP), true negative (TN), and false negative (FN).The QUADAS-2 was applied to systematically evaluate the quality of the studies included in this pooled analysis. With the max QUADAS-2 score of 7, we can judge the quality of the included studies based on the results.

### 2.3. Statistical Analysis

All statistical analyses were performed using Stata 14.0 (Stata, College Station, TX, USA). The pooled sensitivity, specificity, diagnostic odds ratio (DOR), positive likelihood ratio (PLR) and negative likelihood ratio (NLR) and other parameters were calculated by the bivariate meta-analysis model. Then, we performed summary receiver operator characteristic (SROC) curves analysis and calculated the area under the ROC curves (AUC) to assess the overall diagnostic value of lncRNA H19 in cancer detection and diagnosis [30]. These data were confirmed by a hierarchical summary receiver operating characteristics (HSROC) model. Spearman correlation coefficients were conducted to evaluate heterogeneity of threshold effect. Heterogeneity of nonthreshold effects was assessed by Cochran-Q and Inconsistency index (I^2^) test [31]. A P value less than 0.10 for the Q test or I^2^ value higher than 50% indicated obvious heterogeneity between the studies [32]. Moreover, Fagan's Nomogram was used to certify relationships between prior-test probability, likelihood ratio, and posttest probability. The publication bias was tested by Deeks' funnel plots [33].

## 3. Results

### 3.1. Studies Selection and Characteristics of Included Studies

**Figure 1:**
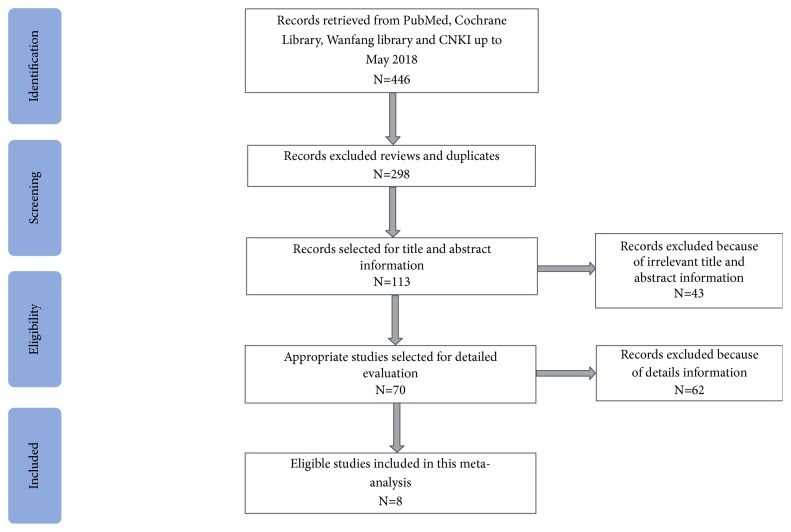
The flow diagram of the included and excluded studies.

By searching PubMed, Web of Science, Wanfang, and CNKI databases, a total of 8 eligible studies [19–26] including 770 cases and 815 controls from 116 records published from 2013 to 2018 were according to inclusion and exclusion criteria (Figure 1). The main features of included articles were displayed in Table 1. In total, there were studies on breast cancer (n=3), gastric cancer (n=3), hepatic carcinoma (n=1), and thyroid cancer (n=1). Among the 8 studies tested lncRNA H19 expression using qRT-PCR methods was based on plasma (n=4), tissue (n=2), serum (n=1), and urinary (n=1).

### 3.2. Quality Assessment

The results of the Quality Assessment of Diagnostic Accuracy Studies-2 (QUADAS-2) study quality assessment were also shown in Table 1. All of the QUADAS-2 scores for studies on diagnosis were ≥4, indicating a moderate-high quality for most of the studies.

### 3.3. Data Analysis

**Figure 2:**
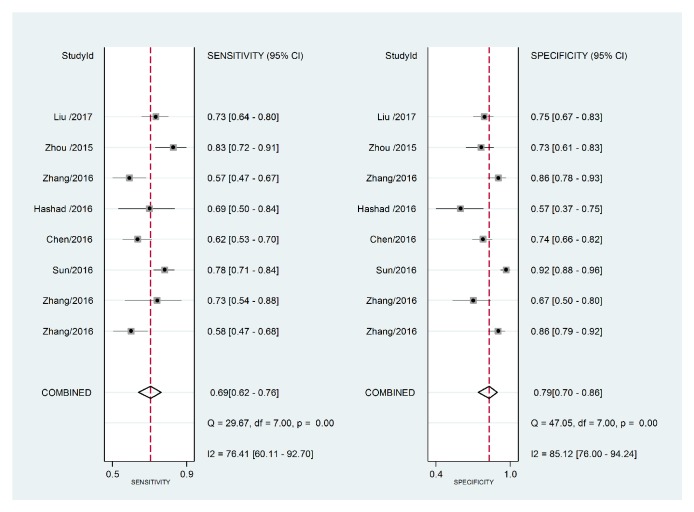
Forest plots of pooled sensitivity and specificity of the overall 8 included publications.

**Figure 3:**
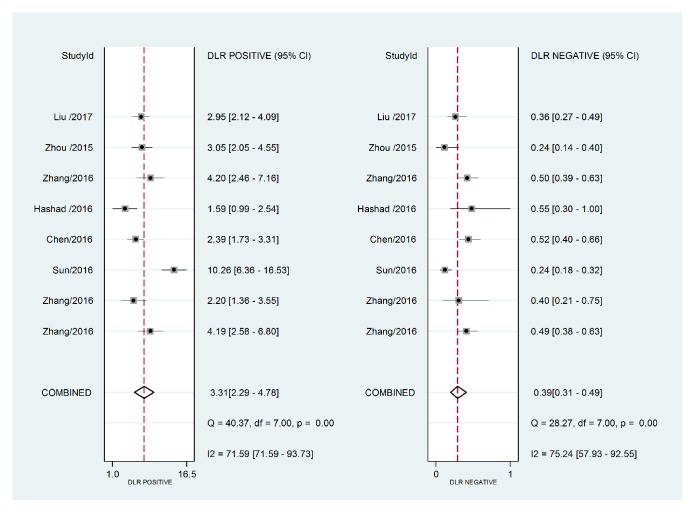
Forest plots of positive likelihood ratio (PLR) and negative likelihood ratio (NLR) for lncRNA H19 in the diagnosis of cancer.

**Figure 4:**
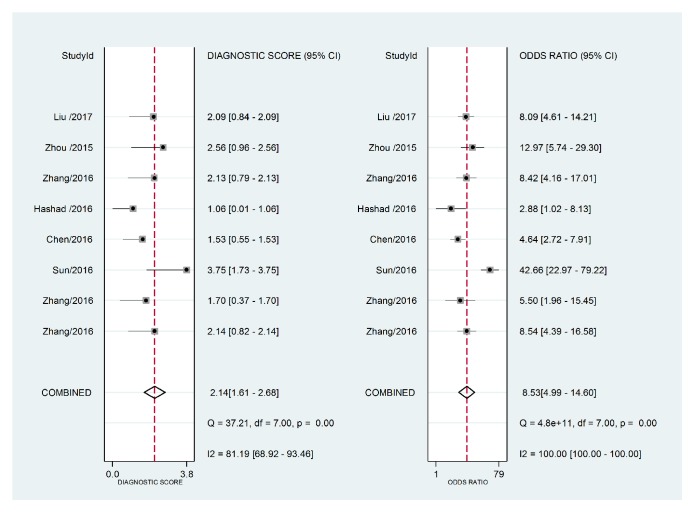
Forest plots of pooled diagnostic odds ratio (DOR) for lncRNA H19 in the diagnosis of cancer.

**Figure 5:**
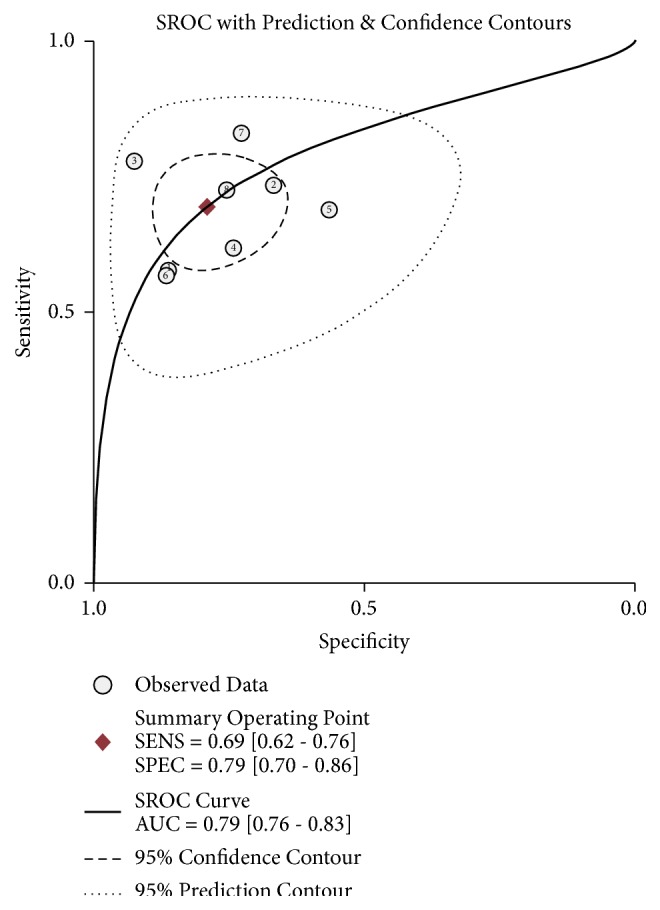
Summary receiver operating characteristic (SROC) graph of included studies.

The forest plot of data from included articles on sensitivity and specificity for H19 assay in diagnosing cancer was shown in Figure 2. Overall, the sensitivity and specificity for the pooled data were 0.69 (95%CI=0.62-0.76) and 0.79 (95%CI=0.70-0.86), respectively. Significant heterogeneity was found for both sensitivity (I^2^=76.41%, 95%CI=60.11%-92.70%) and specificity (I^2^=85.12%, 95%CI=76.00%-94.24%). In addition, the pooled PLR was 3.31 (95%CI=2.29-4.78), the NLR was 0.39 (95%CI=0.31-0.49), and the DOR was 8.53 (95%CI=4.99-14.60) (Figures 3 and 4). The SROC curve for the 8 included studies is shown in Figure 5. The AUC of H19 was 0.79 (95%CI=0.76-0.83), implying a relatively moderate diagnostic value.

**Figure 6:**
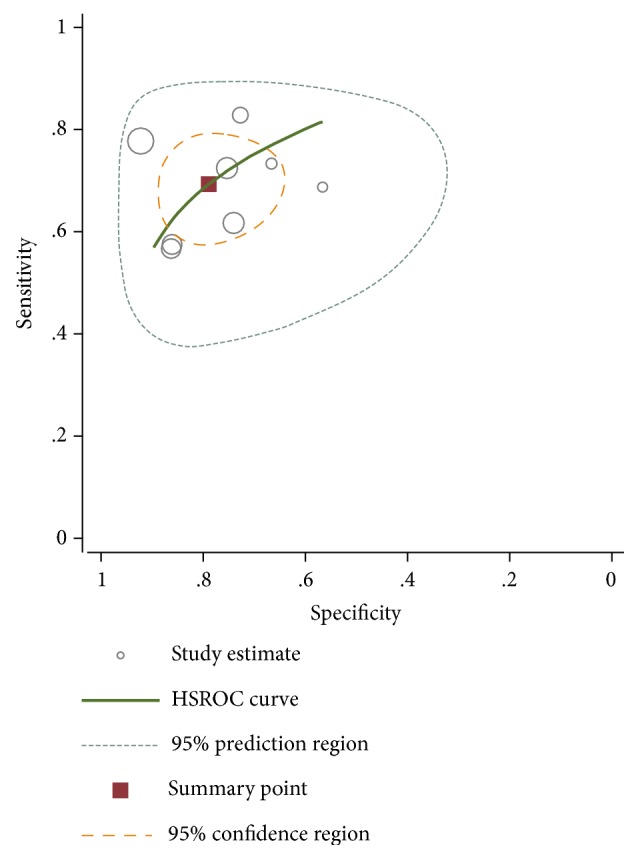
Hierarchical summary receiver operating characteristics (HSROC) curve for lncRNA H19 in the diagnosis of cancer.

**Figure 7:**
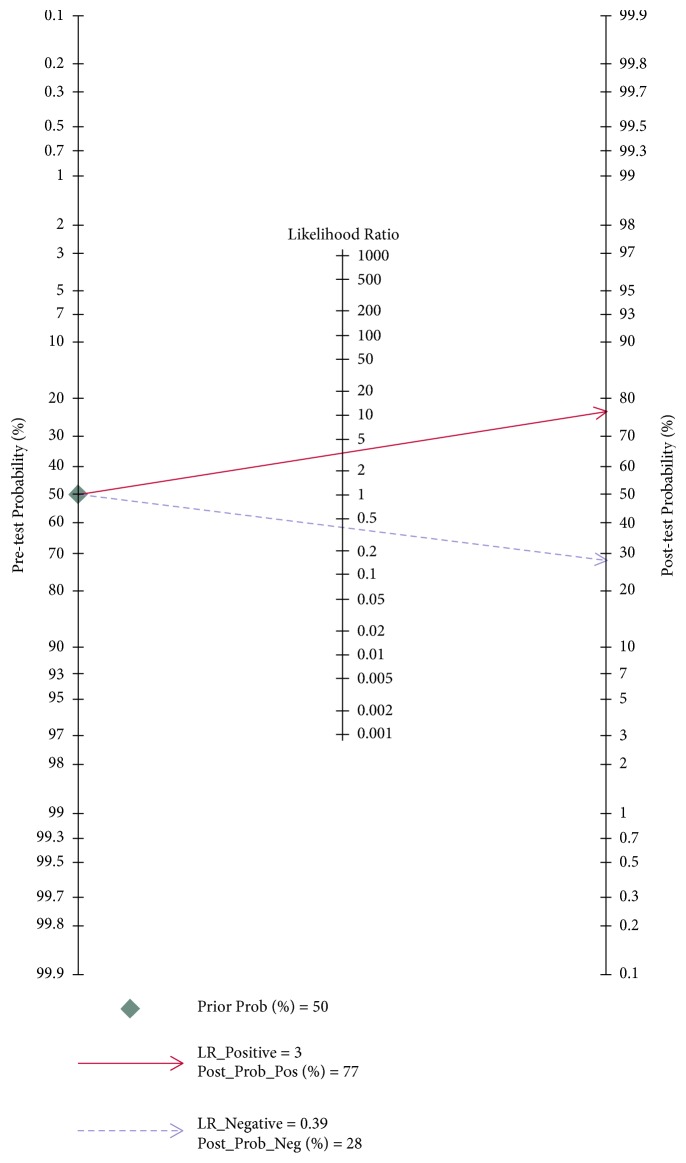
Fagan's Nomogram for calculation of posttest probabilities.

The HSROC curve of these included studies was in line with the results from the bivariate model. The value of *β* was 0.47 (95%CI=0.44-1.39), and the P value was 0.314 which indicated that the HSROC was symmetrical. The value of *γ* was 2.08 (95%CI=1.56-2.60) (Figure 6). To evaluate the clinical utility of the index test, a Fagan's Nomogram was performed to predict the increasing inerrability about a positive diagnosis by using the value of the test and it is used for estimating posttest probabilities. As shown in Figure 7, when H19 assays were tested for all individuals with a pretest probability of 50% to have cancer, a positive result would improve posttest probability having cancer to 77%, while a negative result would drop the posttest probability to 28%. All of the results indicated that H19 had a relatively moderate accuracy in distinguishing cancer patients from all individuals.

### 3.4. Influence Analysis and Robustness Tests

**Figure 8:**
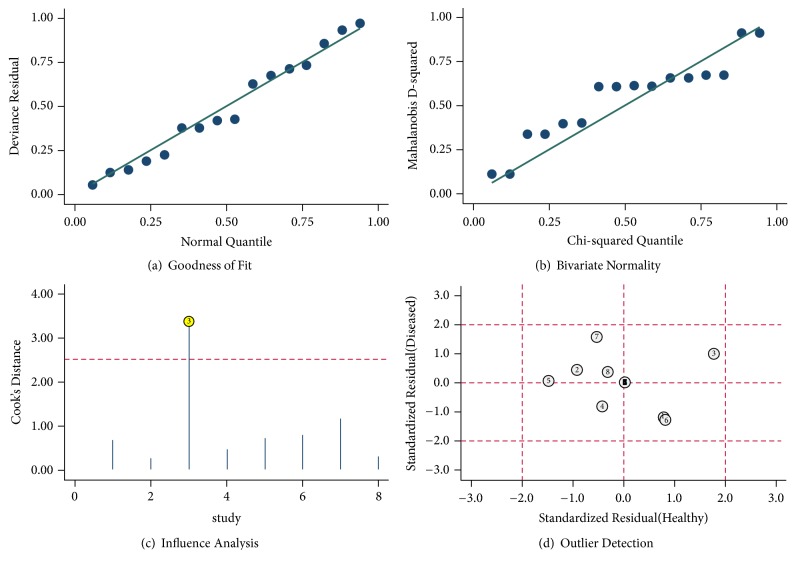
Graphs for sensitivity analyses: (a) goodness of fit, (b) bivariate normality, (c) influence analysis, and (d) outlier detection.

**Figure 9:**
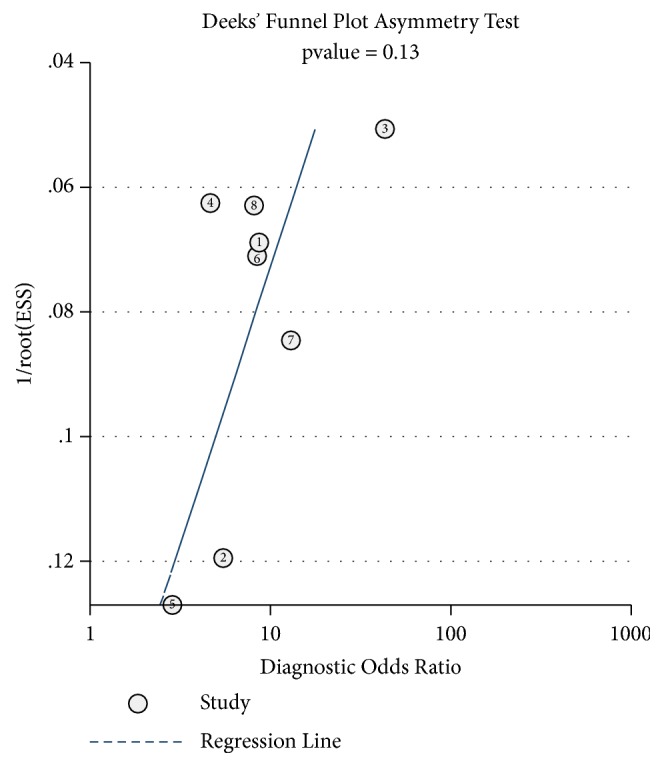
Graph of Deeks' funnel plot asymmetry test.

God-of-fit and bivariate normality analyses (Figures 8(a) and 8(b)) showed that the bivariate model was moderately robust. We also performed sensitivity analyses and further excluded 1 outliner found by influence analysis and outlier detection in Figures 8(c) and 8(d). After exclusion, the sensitivity dropped from 0.69 to 0.68, specificity dropped from 0.79 to 0.76, the PLR dropped from 3.3 to 2.9, the NLR increased from 0.39 to 0.42, DOR dropped from 9 to 7, and AUC decreased from 0.79 to 0.78, showing no significant change after excluding the outliner. Finally, Deeks' funnel plot asymmetry test was conducted to evaluate publication bias in this pooled analysis (Figure 9), which suggested no significant publication bias (P=0.13). The above tests confirm the robustness of our results in present meta-analysis.

### 3.5. Threshold Effect and Heterogeneity

The I^2^ of the heterogeneity test was 89%, indicating significant heterogeneity. In the present study, the calculated Spearman correlation coefficient value was −0.11 with p=0.01 (P<0.05), suggesting that the threshold effect was the major source of heterogeneity. However, there were only eight articles included; metaregression analysis and subgroup analysis cannot be used.

## 4. Discussion

Through the next generation sequencing technology and large-scale transcriptome mapping, many lncRNAs have been reported to involve in the development of cancer as a regulator in a variety of biological processes. These lncRNAs, located in the nucleus, interact with chromatin remodeling complexes (CRCs) to regulate the genes expression locating on the same chromosome in cis or on another chromosome in trans through fine-tuning of chromatin architecture [34, 35]. Previous studies have demonstrated that lncRNAs were associated with tumor proliferation, invasion, replicative senescence, resistance to drugs and radiation by interaction with proteins, RNA, or DNA [36, 37]. Moreover, lncRNAs could serve as diagnosis and prognosis biomarkers in human cancers due to the fact that lncRNAs can be conveniently collected from body fluid, such as plasma and urine [38].

Much effort has been made to investigate the link between aberrant lncRNA expression and cancer, including lncRNA H19 [27–29, 39–41]. Emerging studies have reported that lncRNA H19 was upregulated in various cancers, such as nonsmall cell lung cancer, bladder cancer, breast cancer, and gastric cancer [42–45]. Several studies have been done to explore diagnostic value of lncRNA H19 in cancer detection and diagnosis. However, there are still inconsistent results in diagnostic accuracy and reliability in individual studies. Therefore, we performed this pooled analysis to evaluate the diagnostic value of H19 in cancer detection. The pooled results in the present study were sensitivity of 0.69 (95%CI=0.62-0.76), specificity of 0.79 (95%CI=0.70-0.86), and the AUC of0.79 (95%CI=0.76-0.83), suggesting that H19 may be a potential biomarker to discriminate cancer patients from normal people. In our study, the pooled DOR of 8.53 (95%CI=4.99-14.60) reflects a moderate level of diagnostic accuracy. Additionally, the likelihood ratio (LR) combines the stability of sensitivity and specificity to provide an omnibus index of test performance [46]. In present meta-analysis, a pooled PLR of 3.31 (95%CI=2.29-4.78) and NLR 0.39 (95%CI =0.31-0.49) suggested that patients with cancer have a 3.31-fold higher possibility of being H19 positive for patients with cancer compared with controls, and 39% of all individuals have negative results, implying that the diagnostic value of H19 is relatively moderate. From the Fagan's Nomogram, we found that when a pretest probability of 50% was specified, the posttest probability positivity would raise to 77% with a positive likelihood ratio of 3, and the posttest probability negativity would decrease to 28% with a negative likelihood ratio of 0.39. All of the results revealed that lncRNA H19 had a relatively moderate diagnostic accuracy in cancer detection and diagnosis.

Heterogeneity is an inescapable problem that can interpret the results of the meta-analysis [47]. There was still potential heterogeneity in our present study because of the existence of other confounding factors. In this study, Spearman rank correlation test was performed to analyze the threshold effect, and the Spearman correlation coefficient was -0.11 with p=0.01 (P<0.05), which indicated that threshold effect was a prime source of heterogeneity. In addition, subgroup analysis and metaregression analysis cannot be used because of the insufficient eligible articles. Thus, the possible reasons such as test method and ethnicity were not investigated as sources of heterogeneity.

Nevertheless, several defects of this pooled analysis should be emphasized. First, eight studies with a limited number of subjects were included in this study, which may weaken the reliability for determining the diagnostic value of H19 for different types of cancers. Second, our articles have a very high ratio of data in Chinese populations, which may result in inevitable publication bias. Third, research and sample size in single tumor type was relatively small; more cancer types studies with large sample size need to be included in analysis. Fourth, not all of the studies reported the cutoff values of lncRNA H19. Finally, only publications in English or Chinese were included; researches in other languages should not be missed.

In summary, all of the results indicated that H19 had a relatively moderate accuracy in distinguishing cancer patients from all individuals, suggesting that H19 could serve as a potential diagnostic biomarker for cancer detection and diagnosis. Furthermore, well-designed prospective studies with large sample sizes and different population groups must be conducted in the future to confirm our findings.

## Figures and Tables

**Table 1:** Characteristics of the included studies.

First author	Year	Country	Ethnicity	Cancer type	Normalizer	Sample type	Test method	Cutoff	Cases/ controls	TP	FP	FN	TN	QUADAS-2
Zhang	2016	China	Asian	BRC	*β*-actin	Plasma	qRT-PCR	1.085	97/116	56	16	41	100	5
Zhang	2016	China	Asian	BRC	*β*-actin	Urinary	qRT-PCR	NA	30/42	22	14	8	28	4
Sun	2016	China	Asian	HCC	GAPDH	Serum	qRT-PCR	0. 073	180/211	140	16	40	195	4
Chen	2016	China	Asian	GC	GAPDH	tissue	qRT-PCR	4.615	128/128	79	33	49	95	5
Hashad	2016	Egypt	African	GC	GAPDH	Plasma	qRT-PCR	0.5	32/30	22	13	10	17	6
Zhang	2016	China	Asian	BRC	*β*-actin	Plasma	qRT-PCR	NA	102/96	58	13	44	83	4
Zhou	2015	China	Asian	GC	GAPDH	Plasma	qRT-PCR	NA	70/70	58	19	12	51	5
Liu	2017	China	Asian	TC	GAPDH	Tissue	qRT-PCR	3.58	131/122	95	30	36	92	5

BRC: breast cancer; HCC: hepatic carcinoma; GC: gastric cancer; TC: thyroid cancer; NA.: not available; TP: true positive; FP: false positive; FN: false negative; TN: true negative.
